# Activation of LXR Nuclear Receptors Impairs the Anti-Inflammatory Gene and Functional Profile of M-CSF-Dependent Human Monocyte-Derived Macrophages

**DOI:** 10.3389/fimmu.2022.835478

**Published:** 2022-02-24

**Authors:** Arturo González de la Aleja, Cristina Herrero, Mónica Torres-Torresano, Juan Vladimir de la Rosa, Bárbara Alonso, Enrique Capa-Sardón, Ittai B. Muller, Gerrit Jansen, Amaya Puig-Kröger, Miguel A. Vega, Antonio Castrillo, Ángel L. Corbí

**Affiliations:** ^1^ Myeloid Cell Laboratory, Centro de Investigaciones Biológicas, Consejo Superior de Investigaciones Científicas (CSIC), Madrid, Spain; ^2^ Unidad de Inmuno-Metabolismo e Inflamación, Instituto de Investigación Sanitaria Gregorio Marañón (IiSGM), Madrid, Spain; ^3^ Unidad de Biomedicina (Unidad Asociada al Consejo Superior de Investigaciones Científicas (CSIC)), Instituto Universitario de Investigaciones Biomédicas y Sanitarias (IUIBS), Grupo de Investigación Medio Ambiente y Salud, Universidad de Las Palmas de Gran Canaria, Las Palmas, Spain; ^4^ Department of Clinical Chemistry, Amsterdam University Medical Center, Location VUmc, Amsterdam, Netherlands; ^5^ Department of Rheumatology and Clinical Immunology, Amsterdam University Medical Center, Location VUmc, Amsterdam, Netherlands; ^6^ Instituto Investigaciones Biomédicas “Alberto Sols” (IIBM), and Centro Mixto Consejo Superior de Investigaciones Científicas y Universidad Autónoma de Madrid (Consejo Superior de Investigaciones Científicas (ICSIC)-UAM), Madrid, Spain

**Keywords:** innate immunity, macrophage, macrophage polarization, LXR, inflammation

## Abstract

Liver X Receptors (LXR) control cholesterol metabolism and exert anti-inflammatory actions but their contribution to human macrophage polarization remains unclear. The LXR pathway is enriched in pro-inflammatory macrophages from rheumatoid arthritis as well as in tumors-associated macrophages from human tumors. We now report that LXR activation inhibits the anti-inflammatory gene and functional profile of M-CSF-dependent human macrophages, and prompts the acquisition of a pro-inflammatory gene signature, with both effects being blocked by an LXR inverse agonist. Mechanistically, the LXR-stimulated macrophage polarization shift correlates with diminished expression of MAFB and MAF, which govern the macrophage anti-inflammatory profile, and with enhanced release of activin A. Indeed, LXR activation impaired macrophage polarization in response to tumor-derived ascitic fluids, as well as the expression of MAF- and MAFB-dependent genes. Our results demonstrate that LXR activation limits the anti-inflammatory human macrophage polarization and prompts the acquisition of an inflammatory transcriptional and functional profile.

## Introduction

Macrophages defend the organism against endogenous danger signals and exogenous threats, and initiate and resolve inflammatory responses. To perform these tasks, macrophages can display a huge spectrum of activation (“polarization”) states, whose acquisition depends on their developmental origin, tissue location, and prevailing extracellular cues (1–3). During inflammation, macrophages exert pro-inflammatory and resolving effector functions, whose fine-tuning and sequential occurrence are crucial for tissue injury repair and return to homeostasis. M-CSF and GM-CSF have opposite instructing effects on macrophages during inflammatory responses (4, 5). M-CSF is indispensable for tissue-resident and monocyte-derived macrophage differentiation (6–9), and primes macrophages (M-MØ) for acquisition of an anti-inflammatory and immunosuppressive profile (IL10^high^ TNF^low^ IL23^low^ IL6^low^) (5, 10–19). By contrast, GM-CSF is produced at sites of inflammation (6, 7), and primes macrophages (GM-MØ) for robust antigen-presenting, T cell-stimulatory and pro-inflammatory activity (IL10^low^ TNF^high^ IL23^high^ IL6^high^). Thus, M-MØ resemble tissue-resident ‘trophic’ macrophages, whereas GM-MØ represent pro-inflammatory monocyte-derived macrophages. In line with their effector functions, M-MØ and GM-MØ exhibit distinct transcriptional profiles (10, 13, 20–22) that resemble those of human resident and inflammatory macrophages *in vivo* (20, 23–25), and differ in their responsiveness to the immunosuppressant drug methotrexate (MTX) (26, 27).

LXRα and LXRβ (coded for by *NR1H3* and *NR1H2*, respectively) are ligand-activated transcription factors that regulate gene expression (positively and negatively) in a ligand-dependent manner, and that actively control macrophage differentiation and specialization (28–30). LXR activity is crucially involved in cellular cholesterol metabolism in most tissues. Upon binding of ligand (endogenous cholesterol derivatives, or synthetic agonists like T0901317 and GW3965), LXR positively control the expression of genes that collectively inhibit uptake and promote cholesterol efflux, thus contributing to prevent cellular lipid overload (31–33). LXR ligands also control inflammation in macrophages by antagonizing the induction of inflammation-related genes after activation (29, 34) and potentiating apoptotic cell clearance. These results have led to recognition of LXR as anti-atherogenic and anti-inflammatory factors (35, 36). However, LXR activation exacerbates inflammatory responses in human monocytes, dendritic cells and in a mouse arthritis model (37–40), and agonist-induced LXR activation elicits anti-tumor activity through immune-mediated mechanisms (41), specifically reducing the abundance of immunosuppressive Myeloid-Derived Suppressor Cells (MDSC) and enhancing anti-tumor cytotoxic T lymphocytes (CTLs) activation (41).

Of note, several LXR target genes have been observed within the most enriched pathway in tumor-associated macrophages from colorectal liver metastasis (42), whose presence correlates with a worse prognosis (large TAM) (42, 43). In addition, the LXR pathway is also upregulated in pro-inflammatory macrophages from rheumatoid arthritis (RA) synovial fluid, where LXR activity potentiates cytokine release (44). This apparently discrepancy illustrates the ambiguous contribution of LXR to inflammatory and immune responses, and raises the question of the role of LXR in human macrophage polarization (37, 45). To address this issue, we have now determined the range of LXR target genes in M-MØ (13, 20) and in macrophages generated under the influence of tumor-derived ascitic fluid (TAF-MØ), and assessed the contribution of LXR to the functional capabilities of M-MØ through the use of the established synthetic LXR agonist GW3965 and the inverse agonist GSK2033. We report that LXR activation impairs the acquisition of the transcriptional and functional properties of anti-inflammatory M-MØ and TAF-MØ, and inhibits the expression of the transcription factors (MAF, MAFB) that shape the gene and functional profile of M-CSF-dependent macrophages.

## Materials and Methods

### Generation of Human Monocyte-Derived Macrophages *In Vitro* and Treatments

Human Peripheral Blood Mononuclear Cells (PBMCs) were isolated from buffy coats from anonymous healthy donors (provided by the Centro de Transfusiones de la Comunidad de Madrid) over a Lymphoprep (Nycomed Pharma) gradient according to standard procedures. Monocytes were purified from PBMC by magnetic cell sorting using anti-CD14 microbeads (Miltenyi Biotec). Monocytes (>95% CD14^+^ cells) were cultured at 0.5 x 10^6^ cells/ml in Roswell Park Memorial Institute (RPMI 1640, Gibco) medium supplemented with 10% fetal bovine serum (FBS, Biowest) for 7 days in the presence of 1000 U/ml GM-CSF or 10 ng/ml M-CSF (ImmunoTools) to generate GM-CSF-polarized macrophages (GM-MØ) or M-CSF-polarized macrophages (M-MØ), respectively (46). Cytokines were added every two days and cells were maintained at 37°C in a humidified atmosphere with 5% CO_2_ and 21% O_2_. Where indicated, macrophages were treated at different time points with one dose of LXR agonist GW3965 (31) (1 μM, Tocris), LXR inverse agonist GSK2033 (32) (1 μM, Tocris) or both, using dimethyl sulfoxide (DMSO) as vehicle. In the dual condition, the inverse agonist was added 1-hour prior to agonist treatment. In some experiments, cells were exposed to the GSK3β inhibitor CHIR99021 (2 μM) or DMSO as control. When indicated, Ascitic Fluid from cancer patients (Tumor-derived Ascitic Fluid, TAF) was added to monocytes (0.5:1 in culture medium), and cultures were maintained for 72 h. Ascitic fluids from four metastatic tumors (ovary cancer with peritoneal metastasis, renal carcinoma, and two from gastric carcinoma patients) were kindly provided by Dr. M^a^ Isabel Palomero (Oncology Department, Hospital General Universitario Gregorio Marañón) after patients had provided informed consent (Approval was obtained from the ethics committee of Hospital General Universitario Gregorio Marañón, and the procedures used in this study adhere to the tenets of the Declaration of Helsinki). Samples were centrifuged (4000g, 15 min) to remove cells and particulate material, sterile-filtered, aliquoted, and stored at -80°C until use. The patients provided informed consent and the Hospital General Universitario Gregorio Marañón ethics committee approved the study. For macrophage activation, cells were treated with 10 ng/ml E. coli 055:B5 lipopolysaccharide (Ultrapure LPS, Sigma-Aldrich). Human cytokine production was measured in macrophage supernatants using commercial ELISA [(TNF-α (BD Biosciences), IL-10, Activin A, CCL19, IL1β, IFNβ (R&D Systems)] according to the procedures supplied by the manufacturers.

### Quantitative Real-Time RT-PCR (qRT-PCR)

Total RNA was extracted using the total RNA and protein isolation kit (Macherey-Nagel). RNA samples were reverse-transcribed with High-Capacity cDNA Reverse Transcription reagents kit (Applied Biosystems) according to the manufacturer’s protocol. Real-time quantitative PCR was performed with LightCycler^®^ 480 Probes Master (Roche Life Sciences) and Taqman probes on a standard plate in a Light Cycler^®^ 480 instrument (Roche Diagnostics). Gene-specific oligonucleotides (**Supplementary Table 1**) were designed using the Universal ProbeLibrary software (Roche Life Sciences). Results were normalized to the expression level of the endogenous references genes *TBP*, *HPRT1* or *GAPDH* and quantified using the ΔΔCT (cycle threshold) method.

### Western Blot

M-MØ and GM-MØ cell lysates were subjected to SDS-PAGE (30-50 μg unless indicated otherwise) and transferred onto an Immobilon-P polyvinylidene difluoride membrane (PVDF; Millipore). After blocking the unoccupied sites with 5% non-fat milk diluted in Tris-Buffered Saline plus Tween 20 (TBS-T), protein detection was carried out with antibodies against LXRα (PPZ0412; Biotechne), LXRβ (PPK8917; Biotechne), MAFB (HPA005653, Sigma Aldrich) or c-MAF (sc-7866; Santa Cruz Biotechnology). Protein loading was normalized using an antibody against GAPDH (sc-32233; Santa Cruz Biotechnology) or vinculin (V9131; Sigma-Aldrich). Quimioluminiscence was detected in a Chemidoc Imaging system (BioRad) using SuperSignal™ West Femto (ThermoFisher Scientific).

### RNA-Sequencing and Data Analysis

RNA was isolated from M-MØ generated from monocytes exposed to a single dose of DMSO, GW3965, GSK2033 or both at the beginning of the 7-day differentiation process. Alternatively, RNA was isolated from CD14^+^ monocytes treated with DMSO (vehicle) or 1 μM GW3965 for 1 hour, and then cultured for 3 days in RPMI 1640 with 10% FBS supplemented with 50% Tumor-derived Ascitic Fluid (TAF). Sequencing was done on a BGISEQ-500 platform (https://www.bgitechsolutions.com). RNAseq data were deposited in the Gene Expression Omnibus (http://www.ncbi.nlm.nih.gov/geo/) under accession GSE156783 and GSE181313. On average, 88.04 M reads per sample were generated and clean reads were mapped to the reference (UCSC Genome assembly hg38) using Bowtie2 (average mapping ratio to reference genome, 91.82%) (47). Gene expression levels were calculated by using the RSEM software package (48), and differential gene expression was assessed by using the R-package DESeq2 algorithms using the parameters Fold Change>2 and adjusted p value <0.05. Plots generated with the ggplot2 package, and heatmaps and clustering were done using the Genesis software (http://genome.tugraz.at/genesisclient/) (49). Differentially expressed genes were analyzed for annotated gene sets enrichment using ENRICHR (http://amp.pharm.mssm.edu/Enrichr/) (50, 51), and enrichment terms considered significant with a Benjamini-Hochberg-adjusted p value <0.05. For gene set enrichment analysis (GSEA) (http://software.broadinstitute.org/gsea/index.jsp) (52), gene sets available at the website, as well as gene sets generated from publicly available transcriptional studies (https://www.ncbi.nlm.nih.gov/gds), were used.

### Statistical Analysis

For comparison of means, and unless otherwise indicated, statistical significance of the generated data was evaluated using the paired Student t test in GraphPad Prism 8. In all cases, p<0.05 was considered as statistically significant.

## Results

### Differential Expression of LXRα and LXR-Regulated Genes in Pro-Inflammatory GM-MØ and Anti-Inflammatory M-MØ

**Figure 1:**
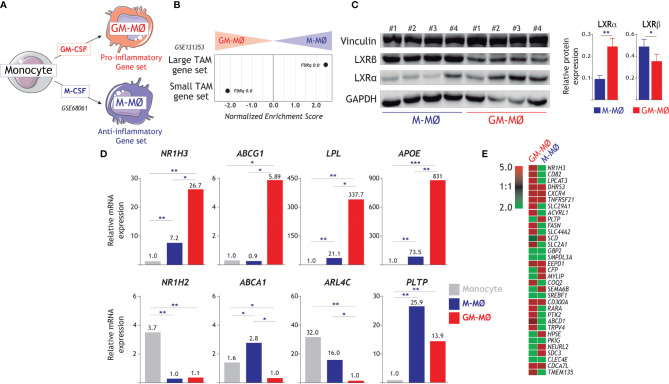
Differential expression of LXR and LXR-regulated genes in GM-MØ and M-MØ. **(A)** Schematic representation of the *in vitro* generation of pro-inflammatory (GM-MØ) and anti-inflammatory (M-MØ) macrophages from peripheral blood human monocytes. **(B)** GSEA of the gene sets that define large TAM and small TAM from colorectal liver metastasis (42) on the ranked comparison of the M-MØ and GM-MØ transcriptomes. Normalized Enrichment Score (NES) and False Discovery rate q value (FDRq) are indicated. **(C)** (Left panel) Protein levels of LXRα and LXRβ in four independent samples (1-4) of GM-MØ and M-MØ, as determined by Western blot, and using vinculin and GAPDH as protein loading controls. (Right panel) LXRα and LXRβ expression in GM-MØ and M-MØ. Mean ± d SEM of 16 independent samples are shown (*p < 0.05; **p < 0.01). **(D)** Relative expression of *NR1H3*, *NR1H2* and the indicated LXR-dependent genes in monocytes, GM-MØ and M-MØ, as determined by RNA-Seq (GSE188278) on 3 independent samples (*, adjp<0.05; **, adjp<10^-5^; ***, adjp<10^-10^). **(E)** Heatmap of the expression of desmosterol-upregulated genes in the gene expression profile of GM-MØ and M-MØ (GSE68061).

We have previously defined gene sets whose expression not only marks human monocyte-derived GM-MØ (“Pro-inflammatory gene set”) and M-MØ (“Anti-inflammatory gene set”) (13, 20) (GSE68061) (**Figure 1A**), but discriminates the gene profiles of pro-inflammatory and immunosuppressive macrophages *in vivo* (24). In fact, the transcriptome of M-MØ shows a very strong enrichment of genes preferentially expressed by large tumor-associated macrophages (large TAM) (**Figure 1B**), whose presence associates with a lower disease-free survival rate in colorectal liver metastasis (42), while the transcriptome of GM-MØ resembles the specific gene profile of “small TAM” (42) and rheumatoid arthritis synovial fluid macrophages (RASF-MØ) (53) (**Figure 1B**). Remarkably, the LXR pathway has been reported to be highly upregulated in large TAM from colorectal liver metastasis (42) but also in pro-inflammatory RASF-MØ (44), thus raising the question of the role of LXR on human macrophage polarization. To clarify this apparent discrepancy, we sought to determine the role of LXR in the generation of monocyte derived macrophages under the influence of either GM-CSF (pro-inflammatory GM-MØ) or M-CSF (anti-inflammatory M-MØ). Initial experiments on a large number of independent samples revealed a higher expression of LXRα (encoded by *NR1H3*) in GM-MØ, and a higher level of *NR1H2*-encoded LXRβ in M-MØ (**Figure 1C**). Besides, RNA-seq (GSE188278) (**Figure 1D**), and the expression of desmosterol-upregulated genes in GM-MØ or M-MØ (54) (**Figure 1E**), showed that the expression of LXR targets is strongly regulated during monocyte differentiation into either GM-MØ or M-MØ (**Figure 1D**), with significantly higher expression of *ABCG1*, *LPL* and *APOE* in GM-MØ, and greater expression of *ABCA1* and *ARL4C* in anti-inflammatory M-MØ (**Figures 1D, E**).

### LXR Activation Shifts the M-CSF-Dependent Differentiation of M-MØ Towards the Pro-Inflammatory Side

**Figure 2:**
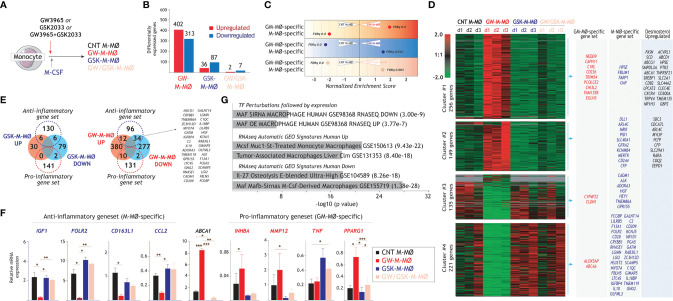
Identification of LXR-regulated genes in anti-inflammatory M-MØ. **(A)** *In vitro* generation of control M-MØ, GW-M-MØ, GSK-M-MØ and GW/GSK-M-MØ before RNA isolation and RNA-sequencing. Control M-MØ were exposed to DMSO in parallel. **(B)** Number of differentially expressed genes (|log_2_FC|>1, adjp<0.05), relative to control M-MØ. **(C)** Summary of GSEA of the “Pro-inflammatory gene set” and “Anti-inflammatory gene set” on the ranked comparisons of the indicated transcriptomes. The color of the circles illustrates the type of enrichment of each comparison (positive, red; negative, blue). The area of each circle is proportional to the Normalized Enrichment Scores of each comparison, which is also indicated. **(D)** Unsupervised clustering of differentially expressed genes (|log_2_FC|>1) between control M-MØ and the transcriptomes of GW-M-MØ, GSK-M-MØ and GW/GSK-M-MØ. For each gene, mRNA expression level in the three donors are represented after normalizing gene expression and k-means clustering using Genesis (http://genome.tugraz.at/genesisclient/). The identification of “Pro-inflammatory gene set”, “Anti-inflammatory gene set” and desmosterol-upregulated genes in each cluster is indicated. **(E)** Comparison of the “Pro-inflammatory gene set” and “Anti-inflammatory gene set” with the genes significantly regulated after M-MØ differentiation in the presence of GSK2033 (left panel) or GW3965 (right panel). **(F)** Relative mRNA expression of the indicated genes from the “Anti-inflammatory” and “Pro-inflammatory” gene sets in GW-M-MØ, GSK-M-MØ and GW/GSK-M-MØ. *ABCA1* expression was evaluated as a readout for LXR activation (*p < 0.05; **p < 0.01; ***p < 0.001). **(G)** Gene ontology analysis of the genes of the “Anti-inflammatory gene set” that are significantly (adjp<0.05) downregulated in GW-M-MØ using Enrichr and the indicated databases.

To directly address the involvement of LXR dependency in the acquisition of the M-MØ transcriptome, monocytes were exposed to a single dose of either the LXR agonist GW3965, the LXR inverse agonist GSK2033 or both, at the beginning of the differentiation process with M-CSF (**Figure 2A**). RNAseq on the resulting macrophages (GW-M-MØ, GSK-M-MØ and GW/GSK-M-MØ) showed that GW3965, and to a lower extent GSK2033, notably altered the acquisition of the transcriptome of M-MØ (**Figure 2B**). GSEA revealed that the most unambiguous effect was observed in GW-M-MØ, whose transcriptome indicated a strong under-representation of the M-MØ-specific “Anti-inflammatory gene set” and a very positive enrichment of the “Pro-inflammatory gene set” (**Figure 2C**). Moreover, comparison of the differentially expressed genes between GW-M-MØ and CNT M-MØ identified a significant number of genes of the “Anti-inflammatory gene set” (with reduced expression in GW-M-MØ, clusters 3 and 4) and the “Pro-inflammatory gene set” (with enhanced expression in GW-M-MØ) (**Figures 2D, E**). Indeed, analysis of a validation set of samples confirmed that differentiation in the presence of GW3965 impairs the expression of genes associated to the anti-inflammatory activities of M-MØ (*IGF1, FOLR2, CD163L1, CCL2*) and augments the expression of paradigmatic genes of the “Pro-inflammatory gene set” (*INHBA*, *PPARG1*) (13, 20, 55, 56) (**Figure 2F**). Conversely, the opposite effects were seen in the case of GSK-M-MØ gene profile (**Figures 2C–F**). Since expression of LXR target genes (e.g., *ABCA1*) (54) confirmed the strong efficacy of GW3965 and GSK2033 in our system (**Figures 2D, F**), these results indicate that LXR activation in monocytes impairs the acquisition of the anti-inflammatory transcriptional profile during the generation of monocyte-derived macrophages in response to M-CSF, and shifts the M-CSF-dependent differentiation of M-MØ towards the pro-inflammatory side. Interestingly, gene ontology analysis (Enrichr) of the 313 genes downregulated in GW-M-MØ showed a strong enrichment in MAF- and MAFB-dependent genes, as well as a highly significant enrichment of the genes that define the profile of “large TAM” from colorectal liver metastasis (42) (GSE131353, **Figure 2G**), thus lending further relevance to the monocyte-conditioning ability of LXR modulators.

**Figure 3:**
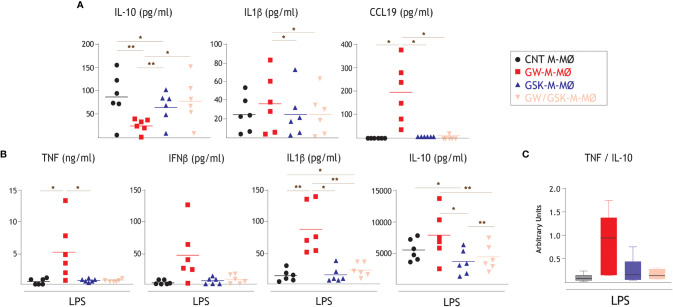
Effect of LXR activation on the cytokine profile of anti-inflammatory M-MØ. **(A)** Production of the indicated cytokines in GW-M-MØ, GSK-M-MØ and GW/GSK-M-MØ, as determined by ELISA. Mean ± SEM of six independent donors are shown (*p < 0.05; **p < 0.01). **(B)** LPS-stimulated (10 ng/ml, 18h) production of TNF, IFNβ1, IL-1β and IL-10 by GW-M-MØ, GSK-M-MØ and GW/GSK-M-MØ, as determined by ELISA. Mean ± SEM of six independent donors are shown (*p < 0.05; **p < 0.01). **(C)** TNF/IL-10 ratio in LPS-stimulated GW-M-MØ, GSK-M-MØ and GW/GSK-M-MØ.

The functional significance of the transcriptional changes observed in GW-M-MØ and GSK-M-MØ was next weighed by comparing their respective cytokine profile in resting conditions and after activation with LPS. Compared to CNT M-MØ, GW-M-MØ produced lower levels of the anti-inflammatory cytokine IL-10 and higher levels of the immuno-stimulatory CCL19 chemokine (**Figure 3A**). Besides, and compared to GW-M-MØ, IL-1β production was significantly lower in GSK-M-MØ and GW/GSK-M-MØ (**Figure 3A**). This pro-inflammatory trend was confirmed after LPS stimulation, as GW-M-MØ secreted higher levels of TNF and IL-1β than CNT M-MØ (**Figure 3B**). Moreover, although LPS-treated GW-M-MØ also secreted higher levels of IL-10, the TNF/IL-10 ratio was considerably higher in GW-M-MØ than in CNT M-MØ (**Figure 3C**). Thus, since the pro-inflammatory effects of GW3965 treatment were impaired or abolished by GSK2033 (GSK-M-MØ and GW/GSK-M-MØ) (**Figures 3A–C**), these results confirm that modulation of LXR activity impacts the inflammatory activity of M-MØ, and that LXR activation prompts monocytes towards the generation of monocyte-derived macrophages with a higher pro-inflammatory transcriptional and cytokine profile.

### The Modulatory Action of LXR Activation Varies Along Monocyte-to-M-MØ Differentiation and Also Affects the Expression of Genes Specifically Associated to M-CSF-Driven Differentiation

**Figure 4:**
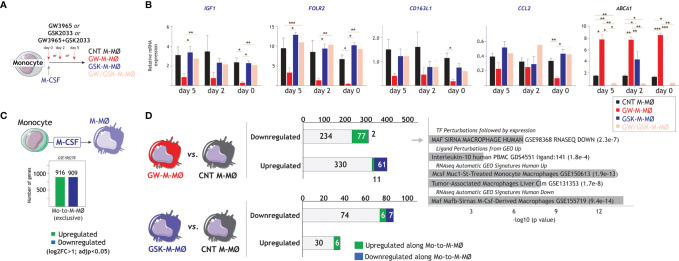
Time-dependency of the effect of LXR activation on the generation of anti-inflammatory M-MØ. **(A)** Schematic representation of the generation of M-MØ after treatment with LXR agonist GW3965 (GW-M-MØ), LXR inverse agonist GSK2033 (GSK-M-MØ) or both (GW/GSK-M-MØ) at distinct long time points during the differentiation process. **(B)** Relative mRNA expression of the indicated genes in M-MØ generated under the indicated treatments. *ABCA1* expression was evaluated as a readout for LXR activation. Mean ± SEM of three independent donors are shown (*p < 0.05; **p < 0.01; ***p < 0.001). **(C)** Schematic representation of the genes specifically upregulated or downregulated along the monocyte-to-M-MØ differentiation (GSE188278). **(D)** (Left panel) Comparison of downregulated or upregulated in GW-M-MØ or GSK-M-MØ with the genes whose expression is upregulated or downregulated along the monocyte-to-M-MØ differentiation. (Right panel) Gene ontology analysis of the genes downregulated in GW-M-MØ and upregulated along the monocyte-to-M-MØ differentiation, using Enrichr and the indicated databases.

Since LXR synthetic ligands cause a chronic and long-lasting modulation of LXR activity, we next assessed their effects at different time points in the monocyte-to-M-MØ differentiation process. To that end, LXR modulators (GW3965, GSK2033, or both) were added at day 0, day 2 or day 5 along M-MØ differentiation (**Figure 4A**), and the expression of the M-MØ-specific “Anti-inflammatory gene set” was determined. As shown in **Figure 4B**, the highest inhibitory effect of GW3965 on the expression of the “Anti-inflammatory gene set” (e.g., *IGF1*, *FOLR2*, *CD163L1*, *CCL2*) was found at the beginning of the differentiation process, and was not significant when added at later time points. By contrast, although the inverse agonist GSK2033 had minor effects, it completely prevented the effect of the agonist at all assayed time points (**Figure 4B**, GW/GSK-M-MØ). These results imply that the involvement of LXR on the M-CSF-dependent acquisition of the transcriptional profile of M-MØ decreases along M-MØ differentiation, being maximal at the start of the monocyte-to-M-MØ differentiation process.

As we have previously identified the genes whose expression is modulated along monocyte-to- M-MØ differentiation (GSE188278) (**Figure 4C**), we next checked whether modulation of LXR activity also affected their expression. As shown in **Figure 4D**, 15% of the genes upregulated in the transcriptome of GW-M-MØ (61 out of 402) were specifically downregulated along monocyte-to- M-MØ differentiation. Conversely, 25% of the genes downregulated in the GW-M-MØ transcriptome (77 out of 313) corresponded to genes specifically upregulated along monocyte-to- M-MØ differentiation (**Figure 4D**). In fact, and in agreement with the analysis of M-MØ-specific genes (**Figure 2**), a very significant percentage of the differentiation-associated genes with reduced expression in GW-M-MØ were MAFB/MAF-dependent, IL-10-regulated, and preferentially found in “large TAM” from colorectal liver metastasis (42) (**Figure 4D**), again emphasizing that LXR activation results in impaired expression of genes directly linked to the MAF/MAFB-dependent generation of anti-inflammatory M-MØ.

### LXR Activity Modulators Alter the Expression of the Factors That Determine the Transcriptional and Functional Profile of M-MØ (MAF, MAFB, Activin A)

**Figure 5:**
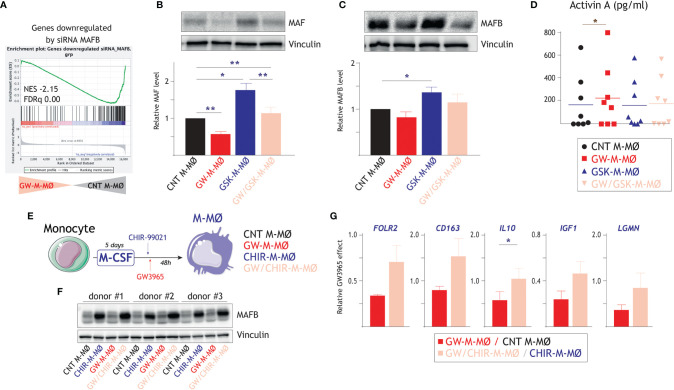
Molecular mechanisms underlying the macrophage polarizing effect of LXR activation. **(A)** GSEA of genes downregulated by siRNA MAFB on the ranked comparison of the transcriptomes of GW-M-MØ and control M-MØ. Normalized Enrichment Score (NES) and False Discovery rate q value (FDRq) are indicated. **(B)** MAF protein levels in GW-M-MØ, GSK-M-MØ and GW/GSK-M-MØ, as determined by Western blot (upper panel) and densitometry (lower panel), and using CNT M-MØ generated in the presence of DMSO as a control. For protein loading control purpose, vinculin protein levels were determined in parallel. (Lower panel). Mean ± SEM of the relative MAF protein levels in the four macrophage subtypes from four independent donors are shown (*p < 0.05; **p < 0.01), and a representative western blot experiment is shown in the upper panel. **(C)** MAFB protein levels in GW-M-MØ, GSK-M-MØ and GW/GSK-M-MØ, as determined by western blot (upper panel) and densitometry (lower panel), and using CNT M-MØ generated in the presence of DMSO as a control. For protein loading control purpose, vinculin protein levels were determined in parallel. (Lower panel). Mean ± SEM of the relative MAFB protein levels in the four macrophage subtypes from four independent donors are shown (*p < 0.05), and a representative western blot experiment is shown in the upper panel. **(D)** Production of activin A in GW-M-MØ, GSK-M-MØ and GW/GSK-M-MØ, as determined by ELISA, and using CNT M-MØ generated in the presence of DMSO as a control. Mean ± SEM of eight independent donors are shown (*p < 0.05). **(E)** Schematic representation of the treatment of differentiating M-MØ with GW3965 in the presence of absence of the GSK3β inhibitor CHIR-99021. **(F)** MAFB protein levels in three independent samples of GW-M-MØ, CHIR-M-MØ, GW/CHIR-M-MØ and CNT M-MØ, as determined by western blot (upper panel). For protein loading control purpose, vinculin protein levels were determined in parallel (lower panel). **(G)** Relative GW3965 effect on the indicated genes in M-MØ generated in the absence (GW-M-MØ vs. CNT M-MØ) or presence (GW/CHIR-M-MØ vs. CHIR-M-MØ) of the GSK3β inhibitor CHIR-99021. Mean ± SEM of three independent donors are shown (*p < 0.05).

MAFB and MAF are master regulators for the differentiation of anti-inflammatory M-MØ (46, 57, 58), whose generation is prevented by activin A (13, 20). Gene ontology analysis using Enrichr (**Figure 2G**) or GSEA (**Figure 5A**) indicate an under-representation of MAF- and MAFB-dependent genes in the transcriptome of GW-M-MØ. Compared to CNT M-MØ, protein analysis revealed that GW-M-MØ has significantly reduced levels of MAF (**Figure 5B**) and MAFB (**Figure 5C**), although the latter did not reach statistical significance. Moreover, GSK-M-MØ expressed higher levels of both MAFB and MAF than CNT M-MØ (**Figures 5B, C**). Altogether, these results agree with the predictions of gene ontology analysis and demonstrate that the pro-inflammatory outcome of LXR activation (GW-M-MØ) correlates with reduced expression of the factors that shape the transcriptional and functional profile of M-MØ, whereas LXR inhibition (GSK-M-MØ) results in enhanced expression of both MAFB and MAF. Furthermore, GW-M-MØ were also found to produce slightly higher levels of activin A (**Figure 5D**), a factor that impairs the differentiation of M-MØ, further supporting that the modulation of LXR activation in monocytes ends up altering the expression of factors that shape the transcriptional and functional profile of M-MØ.

To find out whether MAF/MAFB mediate the macrophage polarizing action of LXR, the expression of both factors was enhanced by inhibiting GSK3β, which controls the stability of large MAF transcription factors through phosphorylation of their transcriptional activation domains (59). Thus, differentiating M-MØ were treated with the GSK3β inhibitor CHIR-99021 before exposure to the LXR agonist GW3965 (**Figure 5E**). As expected, CHIR-99021-treated M-MØ exhibited an enhanced expression of MAFB after 48 hours (**Figure 5F**). More importantly, the GW3965-induced reduction in the expression of M-MØ-specific genes (*FOLR2, CD163, IL10, IGF1, LGMN*) was lower in CHIR-99021-treated M-MØ, an effect that was statistically significant in the case of *IL10* (**Figure 5G**). Therefore, the pro-inflammatory influence of the LXR agonist can be impaired by hindering GSK3β activity, suggesting that MAF and MAFB mediate the inhibitory effect of LXR on the expression of genes of the M-MØ-specific “Anti-inflammatory gene set”.

### LXR Activation Modifies the Polarizing Action of Tumor-Conditioned Medium

**Figure 6:**
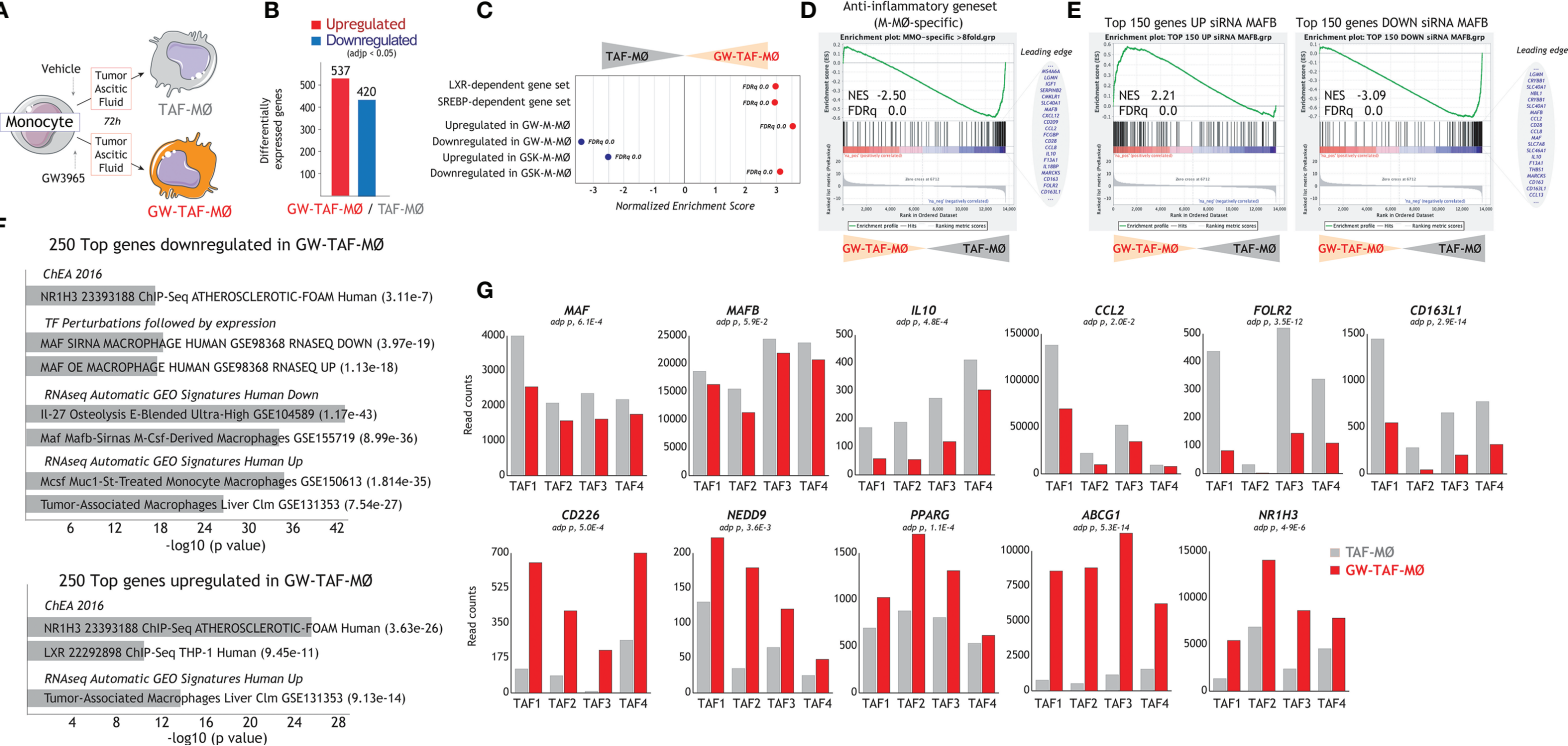
Effect of LXR activation on the transcriptional profile of macrophages generated in the presence of tumor-derived ascitic fluid. **(A)** Schematic representation of the generation of monocyte-derived macrophages in the presence of ascitic fluid from tumor patients, with (GW-TAF-MØ) or without (TAF-MØ) exposure to GW3965. **(B)** Number of differentially expressed genes (adjp<0.05) between GW-TAF-MØ and TAF- MØ. **(C)** Summary of GSEA of the indicated gene sets on the ranked comparison of the GW-TAF-MØ and TAF-MØ transcriptomes. **(D, E)** GSEA of the “Anti-inflammatory gene set” **(D)** and MAFB-regulated gene sets **(E)** on the ranked comparison of the GW-TAF-MØ and TAF- MØ transcriptomes, showing representative genes within the indicated leading edges. In **(C–E)**, Normalized Enrichment Score (NES) and False Discovery rate q value (FDRq) are indicated. **(F)** Gene ontology analysis of the Top 250 genes downregulated (upper panel) or upregulated (lower panel) in GW-TAF-MØ using Enrichr and the indicated databases. **(G)** mRNA expression (RNAseq Read counts) of the indicated genes in GW-TAF-MØ and TAF-MØ generated using four independent tumor-derived ascitic fluids (TAF1-4).

**Figure 7:**
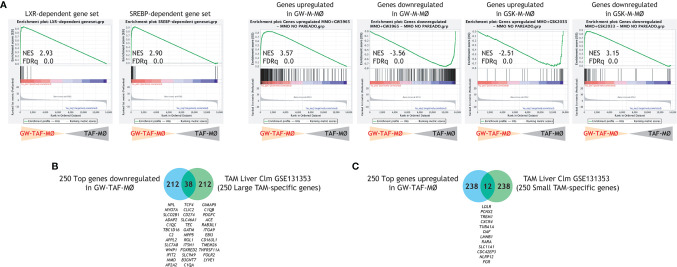
**(A)** GSEA of the indicated gene sets on the ranked comparison of the GW-TAF-MØ and TAF- MØ transcriptomes. Normalized Enrichment Score (NES) and False Discovery rate q value (FDRq) are indicated. **(B, C)** Comparison of the top 250 genes downregulated **(B)** or upregulated **(C)** in GW-TAF-MØ with the genes that characterize the transcriptome of “large TAM” **(B)** or “small TAM” **(C)** from colorectal liver metastasis (42), with indication of overlapping genes.

LXR target gene expression has been previously found to be the most enriched pathway in large TAM from colorectal liver metastasis (42), whose gene profile greatly resembles M-MØ (**Figure 1B**). Thus, to find out the potential relevance of modulating LXR activity under pathological settings, we evaluated whether altering LXR activity was also capable of modifying the macrophage-polarizing ability of tumor-derived ascitic fluids (TAF) of distinct origin. To that end, monocyte-derived macrophages were differentiated in the presence of TAF, and either with or without GW3695 (**Figure 6A**). Comparison of the resulting macrophages (TAF-MØ, GW-TAF-MØ) revealed that LXR activation greatly modifies the transcriptome of macrophages generated under the influence of tumor-derived ascitic fluids, as the expression of almost 1000 genes was significantly altered (**Figure 6B**). As expected, GW-TAF-MØ significantly over-expressed genes regulated by LXR and SREBP (**Figures 6C** and **7A**) as well as of genes upregulated in GW-M-MØ and downregulated in GSK-M-MØ (**Figures 6C** and **7B**). More importantly, GSEA revealed that the gene profile of GW-TAF-MØ shows a very strong under-representation of M-MØ-specific genes (**Figure 6D**) as well as of MAFB-dependent genes (**Figure 6E**), a result further supported by gene ontology analysis using Enrichr (**Figure 6F**). Indeed, and as shown in **Figure 6G**, the expression of representative MAF, MAFB and MAFB-dependent genes was reduced by GW3965 in all GW-TAF-MØ samples, where the expression of GW3965-upregulated genes was higher. Finally, analysis of the transcriptome of GW-TAF-MØ evidenced a very significant downregulation of the genes that define the gene profile of “large TAM” (adjp, 7.54e-27), and a positive enrichment of the genes that characterize “small TAM” (9.13e-14) from colorectal liver metastasis (42) (**Figures 6F** and **7B, C**). These results confirm that LXR activation exerts a similar effect on macrophages generated in the presence of either M-CSF or tumor-derived ascitic fluids, and demonstrate that LXR activation opposes the polarizing action of pathological tumor-derived fluids by impairing the acquisition of the genes that characterize anti-inflammatory (M-CSF-dependent) macrophages and enhancing the expression of genes that define pro-inflammatory macrophages.

## Discussion

LXR nuclear receptors have a prominent role in lipid and cholesterol homeostasis, but also regulate the expression of inflammatory mediators [reviewed in (60)] and are required for the generation of specialized splenic macrophages (30). To clarify the role of LXR in human innate cells, we have analyzed the role of LXR activation in macrophage differentiation and polarization through pharmacological approaches. Our results indicate that LXR has a remarkable influence on the acquisition of the transcriptome that defines anti-inflammatory (M-CSF-dependent) monocyte-derived macrophages, and that LXR over-activation promotes the acquisition of a pro-inflammatory transcriptional and functional profile, whereas abrogation of LXR activity favors the acquisition of anti-inflammatory capacities. Indeed, LXR modulates the cytokine profile of macrophages and modifies their T cell activation activity (not shown), a result that, to our knowledge, has not been previously reported. Mechanistically, modulation of LXR activity led to altered levels of the factors that determine the pro- and anti-inflammatory polarization of human macrophages, namely activin A and MAF/MAFB, respectively. As a whole, our findings reveal a net pro-inflammatory effect of LXR during M-CSF-driven macrophage differentiation. The pathological relevance of our findings is illustrated by the ability of LXR to modulate the macrophage-polarizing action of tumor-derived ascitic fluids.

Numerous studies have evidenced that LXR activation by synthetic ligands suppresses inflammation in the presence of pathogenic stimuli through direct and indirect mechanisms, including transactivation of anti-inflammatory genes and trans-repression of NFκB- and AP1-dependent genes, and *in vivo* results have confirmed the net anti-inflammatory effects of LXR in the presence of macrophage-stimulating agents [reviewed in (60)]. The pro-inflammatory action of LXR that we now report is congruent with those previous studies because we have assayed the effect of LXR activation on unstimulated monocytes that were subsequently exposed to M-CSF. Indeed, it is worth noting that the pro-inflammatory actions of LXR are not unprecedented and have been previously noted (34). In addition to the positive correlation between LXR activation and Rheumatoid Arthritis (38, 39, 44), long term pre-treatment of primary human macrophages to LXR ligands results in potentiated LPS responses (37), and LXR activation leads to increase dendritic cell maturation at the phenotypic, cytokine and functional levels (40). Further, LXR agonists have been recently reported to trigger trained immunity in human monocytes, whereby LXR activation primes macrophages for enhanced responses towards secondary stimuli (45) and impairs MDSC-mediated immunosuppression in cancer (41). While these findings are in line with the enhanced LPS-induced TNF production by GW3965-M-MØ (**Figure 3**), they can also be interpreted as LXR agonist having a macrophage re-polarizing action, because differentiation of M-MØ in the presence of GW3965 results in macrophages with an GM-MØ-like transcriptional and functional profile (enhanced expression of the “Pro-inflammatory gene set”, diminished levels of MAF/MAFB, impaired basal IL-10 production). Globally, all these results illustrate the ability of LXR for macrophage pro-inflammatory re-programming, and suggest that LXR activity modulates inflammatory responses in a cell- or activation-dependent manner, a suggestion that is reminiscent of the macrophage-specific effects of the LXR ligand desmosterol (54).

In the context of the net pro-inflammatory effect of LXR during human monocyte-to-macrophage differentiation, the macrophage re-polarizing action of LXR also shows a high degree of cell-specificity, as the sensitivity to LXR ligands greatly differ among monocytes, differentiating and fully differentiated M-MØ. Altogether, the effect of LXR activity modulators on the expression of polarization-specific genes diminishes along monocyte-to-macrophage differentiation and is inversely correlated with the acquisition of functional polarization. In fact, the sensitivity to LXR modulation at later stages of differentiation is only observed when lower doses of cytokines are used (data not shown). Therefore, the impact of LXR activity on macrophage polarization and functions is more relevant at the monocyte stage and diminishes along macrophage differentiation. The more prominent action of LXR at the beginning of the differentiation model is compatible with a sequential and ordered involvement of different factors, including lineage-determining factors and signal-dependent factors. In our opinion, the potent ligand-activation effect on LXR activity before the M-CSF trigger, unbalances the polarization towards a sustained pro-inflammatory profile. It is possible that activated LXR at the monocyte level promotes its direct binding to regulatory regions of pro-inflammatory and/or epigenetic changes that favor the recruitment of other pro-inflammatory factors.

One possible mechanism underlying the effect of LXR on macrophage pro-inflammatory polarization can be interpreted by our pharmacological analysis. Inhibition of LXR favors the acquisition of an anti-inflammatory transcriptome and leads to a significant increase in the expression of MAF and MAFB, both of which determine the anti-inflammatory polarization of macrophages and the production of IL-10 (46, 58, 61, 62). Whether LXR directly controls the expression of MAF/MAFB in human macrophages would need further investigation. One possible clue came to our attention through the analysis of available datasets from other cell lineages, which confirm the presence of functional LXR-binding sites within GM-MØ-specific genes (“Pro-inflammatory gene set) that are specifically upregulated in the presence of GW3965 (http://cistrome.org/db/#/; CistromeDB: 69799) (63). The link between LXR and MAFB has already been described in the case of the murine osteoclast progenitors, where LXR agonists increased *Mafb* expression through Srebp-1c transactivation of the *Mafb* promoter, without evidence for direct LXR binding to the *Mafb* promoter (64). Therefore, and regardless of the opposite effect of LXR on MAFB in human macrophages and mouse osteoclast progenitors, which might reflect species- or cell-specific differences (65), it appears that LXR is capable of pre-programming myeloid cells by altering the expression of critical regulators of differentiation and polarization.

In summary, we describe a role of LXR nuclear receptors during monocyte-to-macrophage differentiation and in the acquisition of the transcriptional and functional profile of human monocyte-derived macrophages, as LXR activation skews macrophages towards a more pro-inflammatory and stimulatory phenotype. Thus, considering the enhanced activity of LXR in animal models and in human RA (38, 39, 44), modulation of LXR appears as a potential therapeutic target, and LXR-dependent macrophage genes might be considered as useful prognostic/therapeutic markers for human inflammatory diseases. In this regard, it is worth mentioning that, when compared to TAM, RA synovial macrophages display the highest levels of *MMP12*, *INHBA* and *CCL17* (24), whose expression we have now shown to be dependent on LXR. Therefore, it is tempting to speculate that enhancing the level of LXR activation might be beneficial in other pathological settings where the presence of macrophages with deregulated anti-inflammatory and immunosuppressive functions contribute to pathology (e.g., cancer).

## Data Availability Statement

The datasets presented in this study can be found in online repositories. The names of the repository/repositories and accession number(s) can be found below: https://www.ncbi.nlm.nih.gov/geo/, GSE156783, GSE181313.

## Author Contributions

AGA, MT, JR, BA, and EC-S performed research and analyzed data. AGA, AP-K, MV, AC, and ALC designed the research and analyzed data. AGA, AC, and ALC wrote the paper. All authors contributed to the article and approved the submitted version.

## Funding

This work was supported by grants from Ministerio de Ciencia, Investigación y Universidades (SAF2017-83785-R to MV and ALC), Ministerio de Ciencia, Investigación y Universidades y Fondo Europeo de Desarrollo Regional (FEDER) (SAF2017-90604-REDT and PID2019-104284RB-I00/AEI/10.13039/501100011033 to AC), Fundación La Marató/TV3 (Grant 201619.31 to ALC), Instituto de Salud Carlos III (Grant PI20/00316 to AP-K), and Red de Investigación en Enfermedades Reumáticas (RIER, RD16/0012/0007) from Instituto de Salud Carlos III and cofinanced by the European Regional Development Fund “A way to achieve Europe” (ERDF) to AP-K and ALC. This work was also supported in part by a grant from the Dutch Society for Clinical Chemistry (NVKC) to IM and R. de Jonge. AGA was funded by FPU predoctoral fellowship (FPU16/02756) from Ministerio de Universidades.

## Conflict of Interest

The authors declare that the research was conducted in the absence of any commercial or financial relationships that could be construed as a potential conflict of interest.

## Publisher’s Note

All claims expressed in this article are solely those of the authors and do not necessarily represent those of their affiliated organizations, or those of the publisher, the editors and the reviewers. Any product that may be evaluated in this article, or claim that may be made by its manufacturer, is not guaranteed or endorsed by the publisher.
